# 588. A 10-year Review of the Geriatric Burn Population at a Single Burn Center

**DOI:** 10.1093/jbcr/irag033.361

**Published:** 2026-04-06

**Authors:** Morgan M C Karlok, Lori Chrisco, Felicia N Williams, Booker King, Alexandra R Coward, Jamie L Hollowell, Mu Jin, Chris B Agala

**Affiliations:** University of North Carolina, Cary, NC; University of North Carolina, Durham, NC; University of North Carolina, Chapel Hill, NC; University of North Carolina, Durham, NC; University of North Carolina, Durham, NC; North Carolina Jaycee Burn Center, Chapel Hill, NC; University of North Carolina, Chapel Hill, NC; University of North Carolina Chapel Hill School of Medicine Surgery Department, Chapel Hill, NC

## Abstract

**Introduction:**

Burn injuries in the elderly are associated with more severe injuries, longer lengths of stay, and poor outcomes. In 2020, more than 16% of the United States population was 65 years of age or older, and this number is only expected to increase. As elderly patients often have increased frailty, reduced independence, and cognition changes, they are especially vulnerable after any injury, including burns. We sought to identify and describe trends in geriatric burn patients at our regional burn center.

**Methods:**

We conducted a retrospective analysis of adult burn patients admitted between 2015 and 2024 using burn registry data stratified by geriatric status (≥65 years). Demographic and clinical characteristics were compared between age groups using chi square or Fisher’s exact tests and t-test or Wilcoxon rank sum tests. Bivariate and multivariable linear and Poisson regression models were used to assess temporal trends and evaluate associations between calendar year and outcomes (patient age, percent total body surface area [TBSA], and length of stay [LOS], mortality and number of comorbidities).

**Results:**

Among 8852 burn patients, 1367 (15.4%) were ≥ 65 years. Across the 10-year period, mean age at admission increased by 0.48 years per calendar year (95% CI: 0.35-0.61; *p*<.001). Males accounted for 64.6% (n = 833) of geriatric patients. The most common mechanism of injury was flame burns (n = 817) followed by scalds (n = 377). The average TBSA burned was 6.8%. Average TBSA showed an upward trend, increasing by 0.35% per year after adjustment (95% CI: 0.14-0.56; *p*=.001). Comorbidities were common: 86.5% (n = 1182) of patients had at least one comorbidity, while 65.2% (n = 891) had two or more. Intensive care unit (ICU) admission was required for 28.9% (n = 395) of patients, with an average ICU length of stay of 13.2 days. Average hospital length of stay was 13.7 days, with a mean of 4.3 hospital days per percent TBSA burned. In-hospital mortality rate was 8.8% (n = 120). Among survivors, there was a significant increase in the number of patients discharged home (*p*=.049), with 74.2% (n = 1014) discharged directly home.

**Conclusions:**

As the population ages, burn injuries among the elderly are expected to increase. The elderly have increased risk of mortality at baseline and often present with comorbid conditions that further complicate all phases of burn care. The need for age-appropriate care is essential to optimize their treatment and recovery. By identifying and understanding trends in geriatric burn injuries, we are better equipped to prevent and treat this vulnerable population.

**Applicability of Research to Practice:**

Understanding trends in geriatric burn injuries enables burn centers to tailor age-appropriate care strategies for an increasingly vulnerable and complex patient population.

**Funding for the study:**

N/A.

